# A Full Digital Workflow for Masking Stained Teeth Using Ceramic Cores and Veneers

**DOI:** 10.1002/ccr3.72773

**Published:** 2026-05-22

**Authors:** Francisco Garcia‐Torres, Enrico Steger, Alexander Lichtmannegger, Carlos A. Jurado, Silvia Rojas‐Rueda, Franciele Floriani

**Affiliations:** ^1^ Department of Prosthodontics University of La Salle Bajio School of Dentistry Leon Mexico; ^2^ Zirkonzahn GmbH Gais Italy; ^3^ Ponce Health Sciences University School of Dental Medicine Ponce Puerto Rico; ^4^ Department of Prosthodontics The University of Iowa College of Dentistry Iowa City Iowa USA

**Keywords:** CAD/CAM, ceramic veneer, digital dentistry, esthetic rehabilitation, intraoral scanning, intrinsic tooth discoloration

## Abstract

This clinical case report describes a fully digital workflow for managing intrinsic tooth discoloration in the maxillary right central incisor. The approach involved masking the dark underlying substrate with a ceramic core, followed by a veneer restoration. To enhance symmetry and gingival contour, a gingivectomy was performed, and a veneer was also placed on the maxillary left central incisor. The treatment began with an intraoral scan, digital wax‐up, and fabrication of interim restorations, which guided the gingivectomy procedure. After healing, new intraoral and facial scans were performed to digitally design and mill a ceramic core for the right central incisor. Once the core was cemented, final scans were obtained to design and fabricate the definitive veneers. All restorations were bonded under rubber dam isolation to prevent contamination and optimize adhesion. This clinical report demonstrates that using a ceramic core effectively masks dark internal discoloration, allowing the fabrication of highly esthetic veneers with natural contours. The integration of intraoral and facial scanning technologies enables an accurate, reproducible, and efficient digital workflow that enhances both precision and esthetic outcomes in anterior restorations.

## Key Clinical Message

A fully digital workflow combined with the use of a ceramic core provides a predictable and effective approach for masking intrinsic discoloration and achieving optimal esthetic outcomes in anterior restorations.

## 1 Introduction

The demand for esthetic dentistry procedures has significantly increased over the past few decades due to factors such as the influence of social media, greater emphasis on appearance and self‐image, and advancements in less‐invasive and more affordable cosmetic treatments [1, 2]. It has been demonstrated that good oral health—including an attractive smile—can significantly affect self‐confidence and sexual well‐being in young adults, ultimately impacting overall quality of life [3]. One of the primary sources of dissatisfaction with a person's smile is related to the shade, size, arrangement, and position of the teeth [4]. These concerns can often be addressed through orthodontic and/or restorative dental treatments.

In restorative dentistry, all‐ceramic restorations have gained popularity and are increasingly becoming the first choice for restoring the anterior dentition, replacing traditional porcelain‐fused‐to‐metal (PFM) restorations. All‐ceramic options eliminate the grayish discoloration often seen in metal‐ceramic restorations [5]. Studies have reported that the top three material choices for anterior restorations are lithium disilicate, followed by layered zirconia and leucite‐reinforced ceramics [6]. All‐ceramic crowns in the anterior region have demonstrated excellent long‐term success and low failure rates [7], while all‐ceramic veneer restorations in the esthetic zone have shown survival rates exceeding 90% over periods longer than 10 years [8].

Ceramic veneer restorations have been shown to successfully fulfill patients' esthetic demands [9]. However, they pose a challenge when teeth present stains because a ceramic veneer may not be able to block out the dark background [10]. In some clinical situations, tooth whitening can be performed before veneer placement, but whitening does not always achieve the desired shade. Traditionally, full‐coverage crowns were recommended when a dark background is present [11, 12]. More recently, novel approaches have been suggested, such as placing an all‐ceramic core to mask the stain, followed by a veneer restoration [13].

The combination of a ceramic core and veneer may offer clinicians a way to successfully mask dark tooth shade and enable dental technicians to fabricate highly esthetic restorations. Unfortunately, clinical reports in the literature are very limited, and the procedural steps can be confusing for less experienced clinicians. Therefore, this report presents a case in which a severely internally stained maxillary right central incisor was treated with a ceramic core and lithium disilicate veneer to meet the patient's esthetic expectations. The multidisciplinary treatment also included gingivectomy to improve the gingival architecture of that incisor, and a single ceramic veneer on the left central incisor to address incisal and facial wear.

## 2 Case History/Examination

**FIGURE 1:**
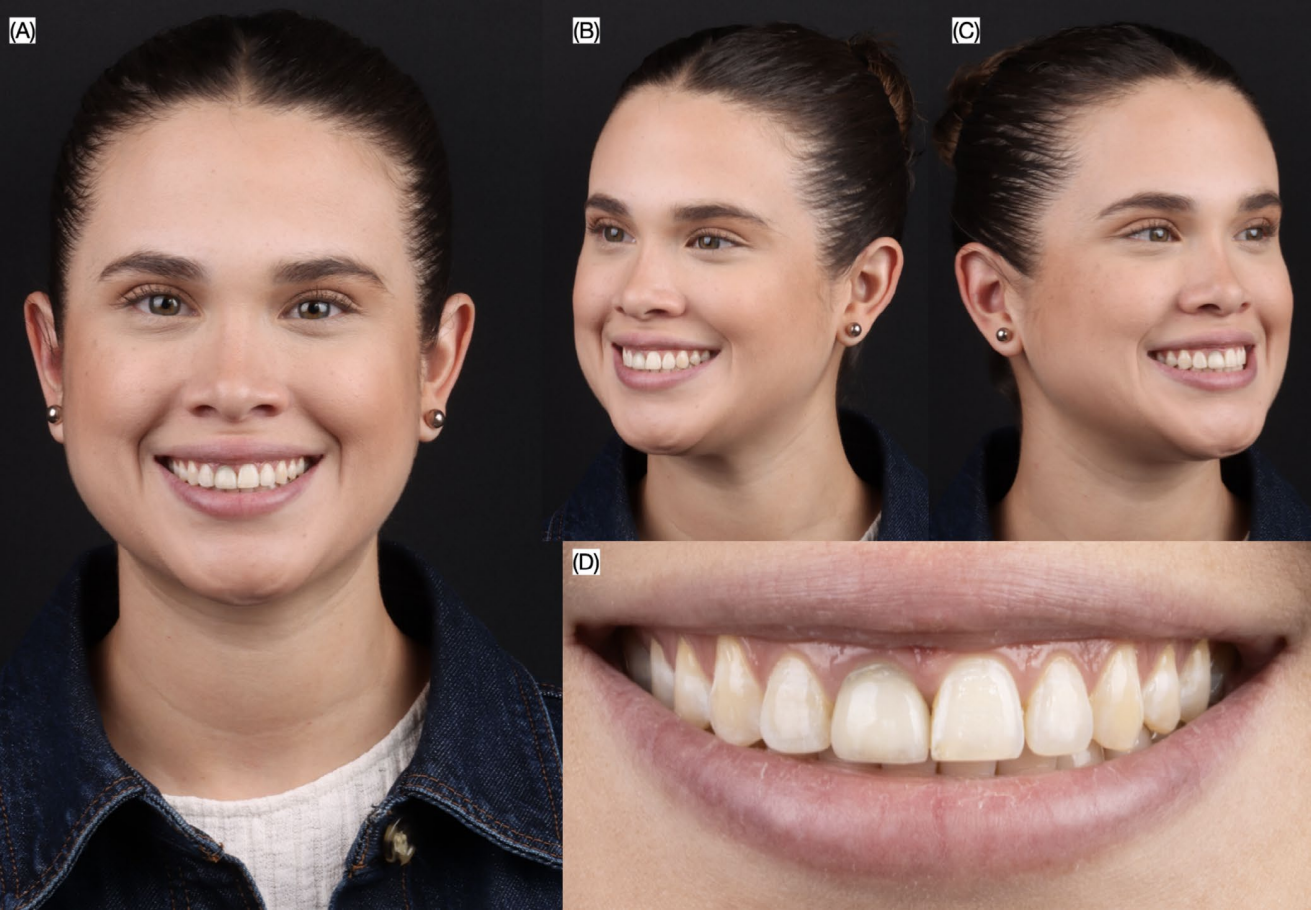
Initial situation. Face smiling (A) frontal, (B) left, and (C) right side view, and (D) close‐up of the smile.

**FIGURE 2:**
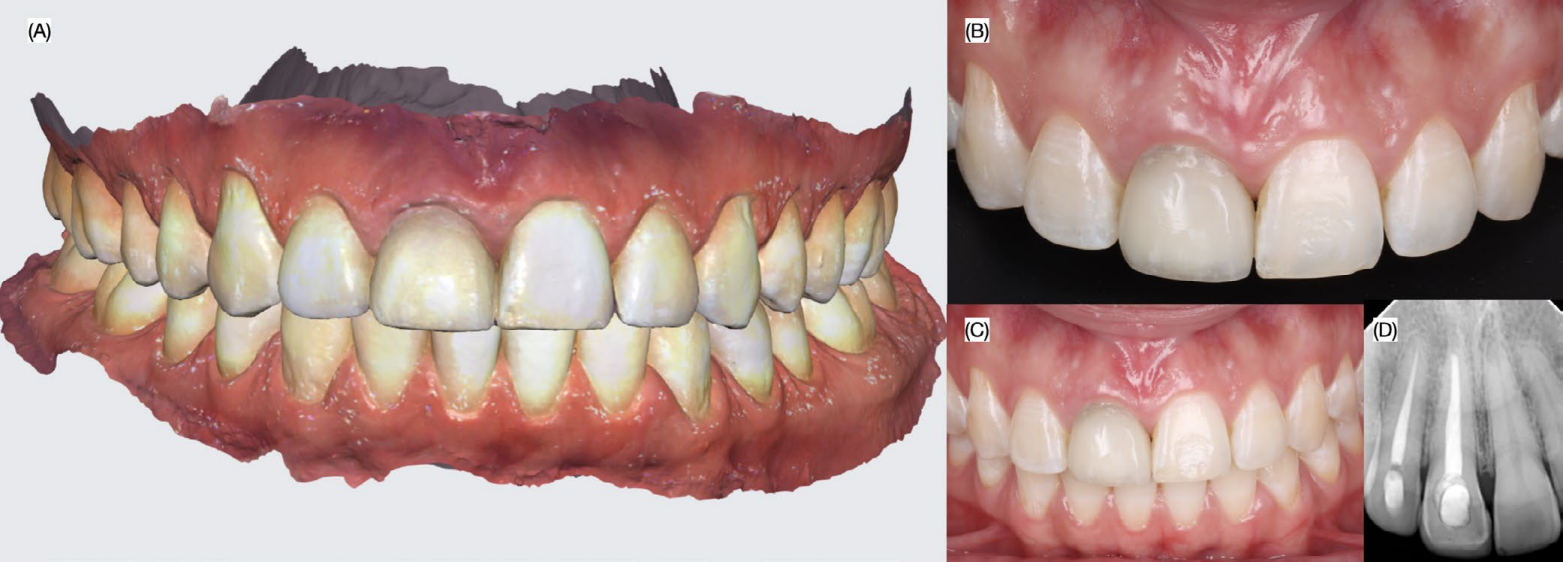
Intra‐oral evaluation. (A) Intra‐oral scan, (B) view of anterior teeth, (C) in occlusion and (D) radiograph.

A 27‐year‐old female patient presented to the dental clinic with the chief complaint: “I do not like my dark front tooth” (Figure 1). She reported receiving endodontic therapy and a porcelain veneer on the maxillary right central incisor 6 years earlier after injuring the tooth while playing basketball. Over time, the tooth began to exhibit dark staining, which had become a significant esthetic concern for her. The patient denied taking any medications or having any allergies and reported being in good general health. She was therefore classified as ASA I. Clinical and radiographic evaluation revealed Class I occlusion and an endodontically treated maxillary right central incisor restored with a porcelain veneer. A gray stain was visible in the middle and gingival third of the restoration. Additionally, the gingival contour of the right central incisor was less ideal than that of the adjacent left central incisor, showing a lower gingival height. A black triangle was present between the central incisors, and incisal and facial wear was noted on the maxillary left central incisor. The gingival zeniths of the left central and lateral incisors were at the same level. Periodontal probing revealed a 4 mm pocket depth on the maxillary right central incisor (Figure 2). A clinical timeline was established to document the sequence of treatment: initial presentation and diagnosis; intraoral and facial scanning with digital wax‐up; fabrication of interim restorations; gingivectomy procedure; tooth preparation; design and milling of the zirconia core; core cementation; definitive veneer fabrication and bonding; and periodic follow‐up, including evaluation at 2 years.

## 3 Differential Diagnosis, Investigation and Treatment

Several treatment options were proposed to the patient. These included crown lengthening to enhance the gingival architecture, followed by the replacement of the current veneer with a ceramic core and veneer restoration on the maxillary right central incisor, along with a matching veneer on the left central incisor. The patient was informed that crown lengthening could significantly improve the esthetic gingival contours. She was also advised that a ceramic core could effectively mask the dark internal staining of the right central incisor, creating a more uniform substrate to match the adjacent tooth, and that placing veneers on both central incisors would provide a more symmetrical esthetic result. The patient declined crown lengthening but agreed to undergo a gingivectomy on the maxillary right central incisor to help reduce the discrepancy in gingival zenith levels. She also consented to the placement of a ceramic core and new veneers on both central incisors. Finally, the option to perform the treatment using a fully digital workflow was discussed. The patient agreed to proceed with this approach, allowing for digital design and fabrication of the restorations.

**FIGURE 3:**
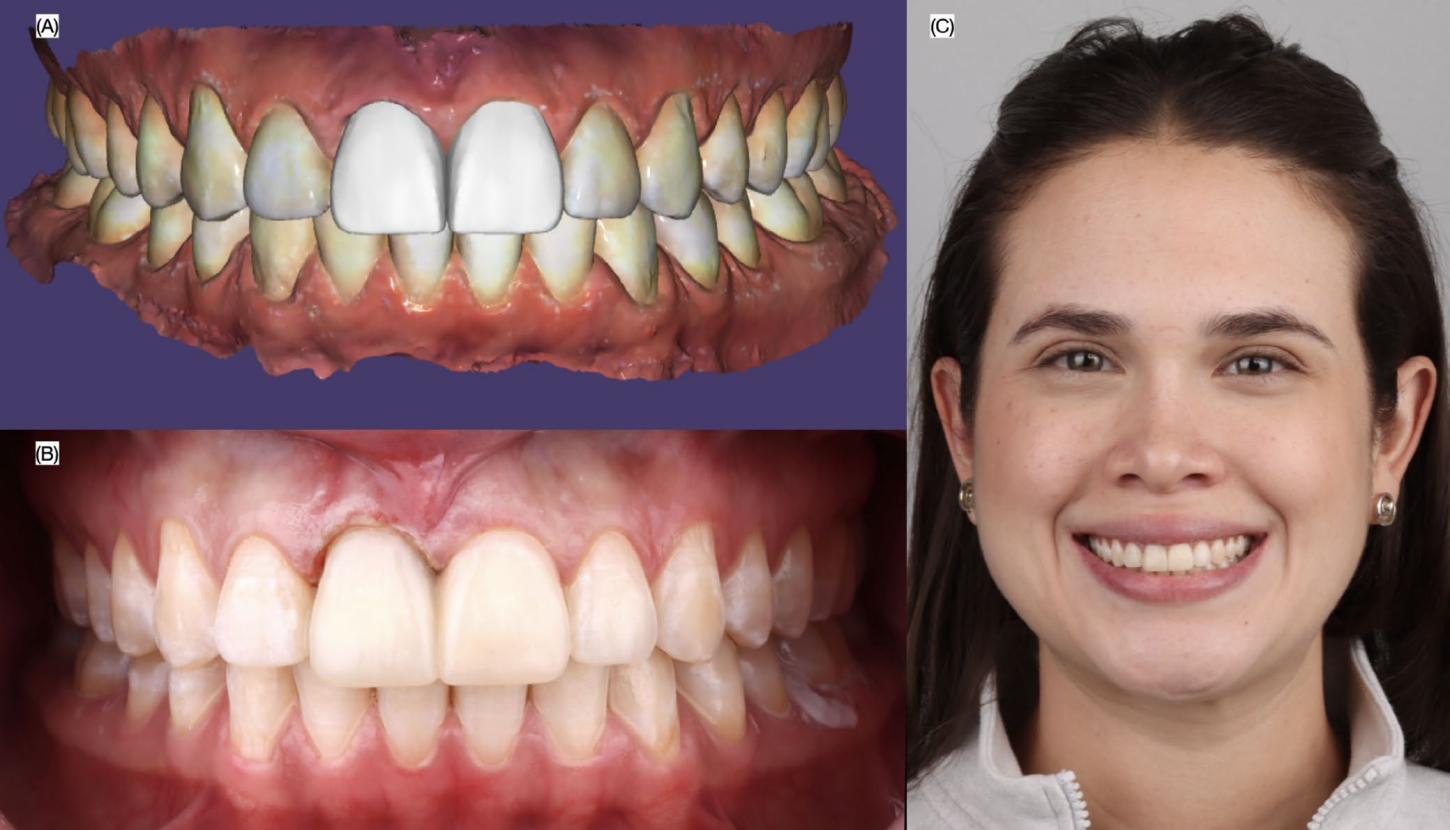
Gingivectomy. (A) Digital planning of the gingivectomy, (B) provisional as a guide for the gingivectomy, and (C) face smiling.

**FIGURE 4:**
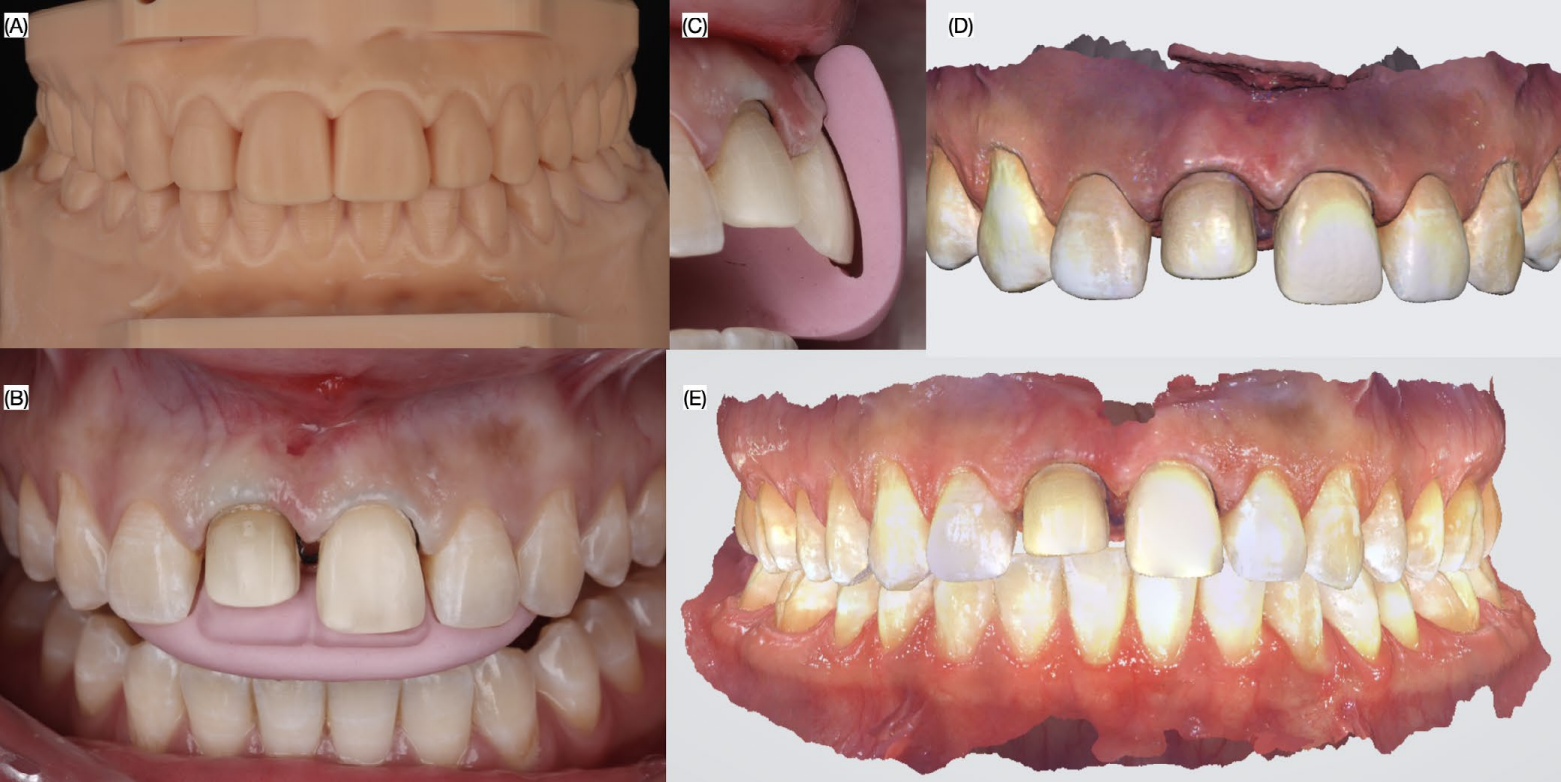
Tooth preparations. (A) Printed model with the desired contours, conservative tooth preparations; (B) frontal and (C) lateral views; and the final digital impression (D) frontal view and (E) in occlusion.

An intraoral scan (Medit i700, Medit Corp, Seoul, South Korea) was performed, followed by a digital wax‐up (DentalCAD, Exocad GmbH, Darmstadt, Germany) for veneers on both central incisors. The design included a 2 mm apical adjustment of the zenith for the maxillary right central incisor. A 3D model incorporating the wax‐up was printed (Die and Model 2, SprintRay, Los Angeles, CA, USA), and a mock‐up putty index was fabricated (Zetalabor, Zhermack SpA, Badia Polesine, Italy). An intraoral mock‐up (Structur Premium, Voco GmbH, Cuxhaven, Germany), was conducted, and its contours were used to guide the clinician during gingivectomy with an electrosurgical unit (The Electron Art‐E1, BonART Co Ltd., Keelung City, Taiwan; Figure 3). After a six‐week healing period, the patient returned for tooth preparation. A double‐zero retraction cord (Ultrapak Retraction Cord, Ultradent Products Inc., South Jordan, UT, USA) was placed, and reduction guides (Zetalabor, Zhermack SpA, Badia Polesine, Italy) were used to perform minimally invasive preparations. The maxillary left central incisor received a conservative 0.5 mm veneer preparation using a specialized kit (M‐I‐R‐A Kit, Jota AG, Rüthi, Switzerland). The existing ceramic veneer on the maxillary right central incisor was removed, and a full crown preparation was completed, with 1.0 mm facial and lingual reduction and a chamfer finish line. To ensure a predictable masking of the underlying dark substrate, a full‐coverage zirconia coping was designed as a ceramic core for the maxillary right central incisor. The coping was digitally planned to provide uniform and controlled thickness, with target values of approximately 0.6 mm in the cervical third, 0.7 mm in the middle third, and 0.8 mm in the incisal third. This thickness gradient was selected to balance optimal masking ability with adequate space for the overlying veneer and to preserve natural tooth contours. The zirconia material was selected based on its high opacity and masking capacity, while still allowing sufficient translucency to avoid an overly opaque or artificial appearance. A medium‐opacity zirconia was chosen to achieve an appropriate compromise between esthetic integration and substrate masking. The definitive veneer was designed with a thickness ranging from approximately 0.5 mm in the cervical third, 0.6 mm in the middle third, and up to 0.8 mm in the incisal third to enhance incisal translucency and optical depth. All thickness parameters were digitally controlled using CAD software based on the initial digital wax‐up, allowing precise standardization and reproducibility of the restorative design. This approach ensured consistent space allocation for both the core and veneer, optimizing both functional and esthetic outcomes. A final digital impression was taken using the (Medit i700, Medit Crp, Seoul, South Korea; Figure 4).

**FIGURE 5:**
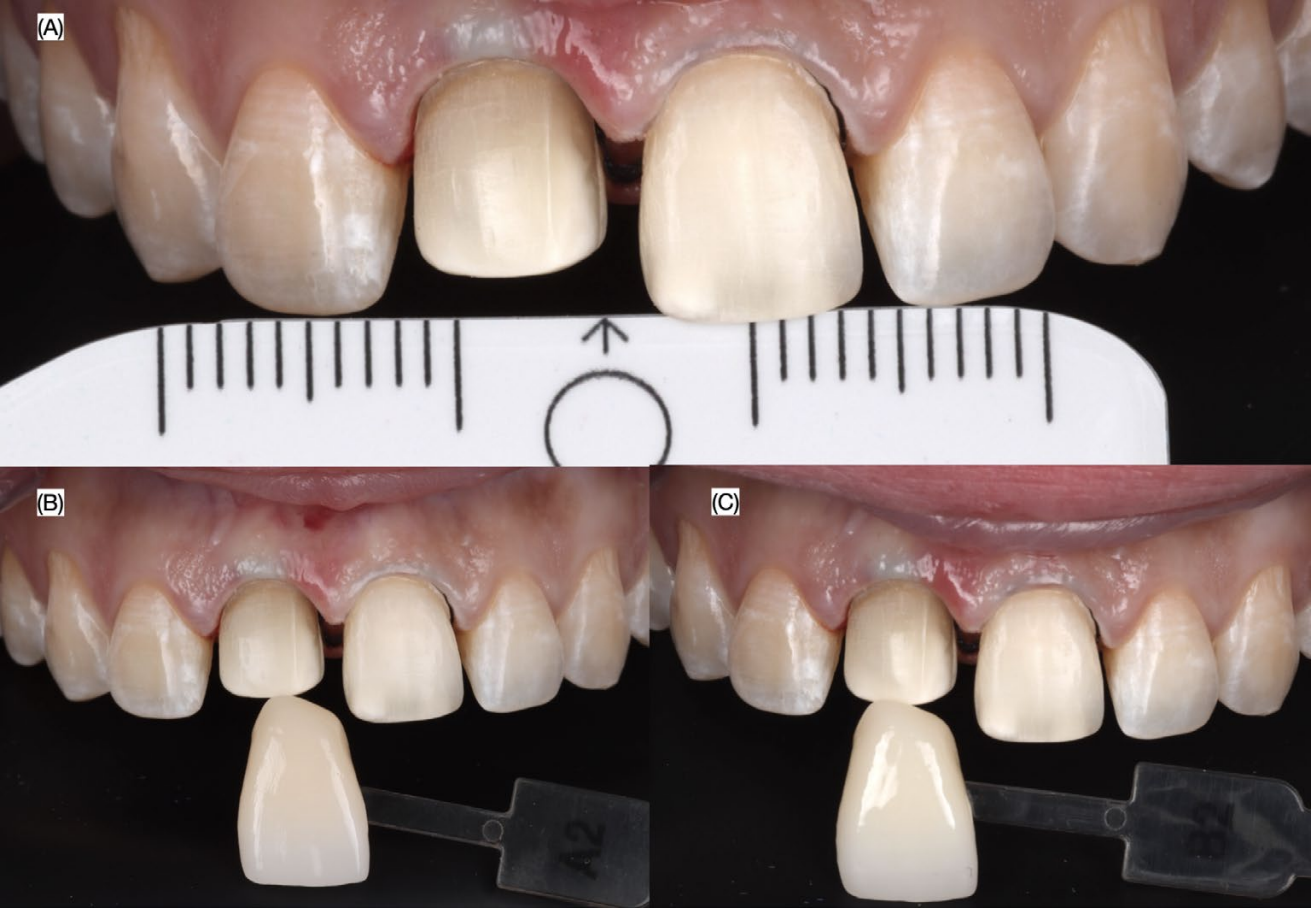
Shade guide photos. (A) Frontal photo with a photo calibration card, and with (B) A2 and (C) B2 shade tabs.

**FIGURE 6:**
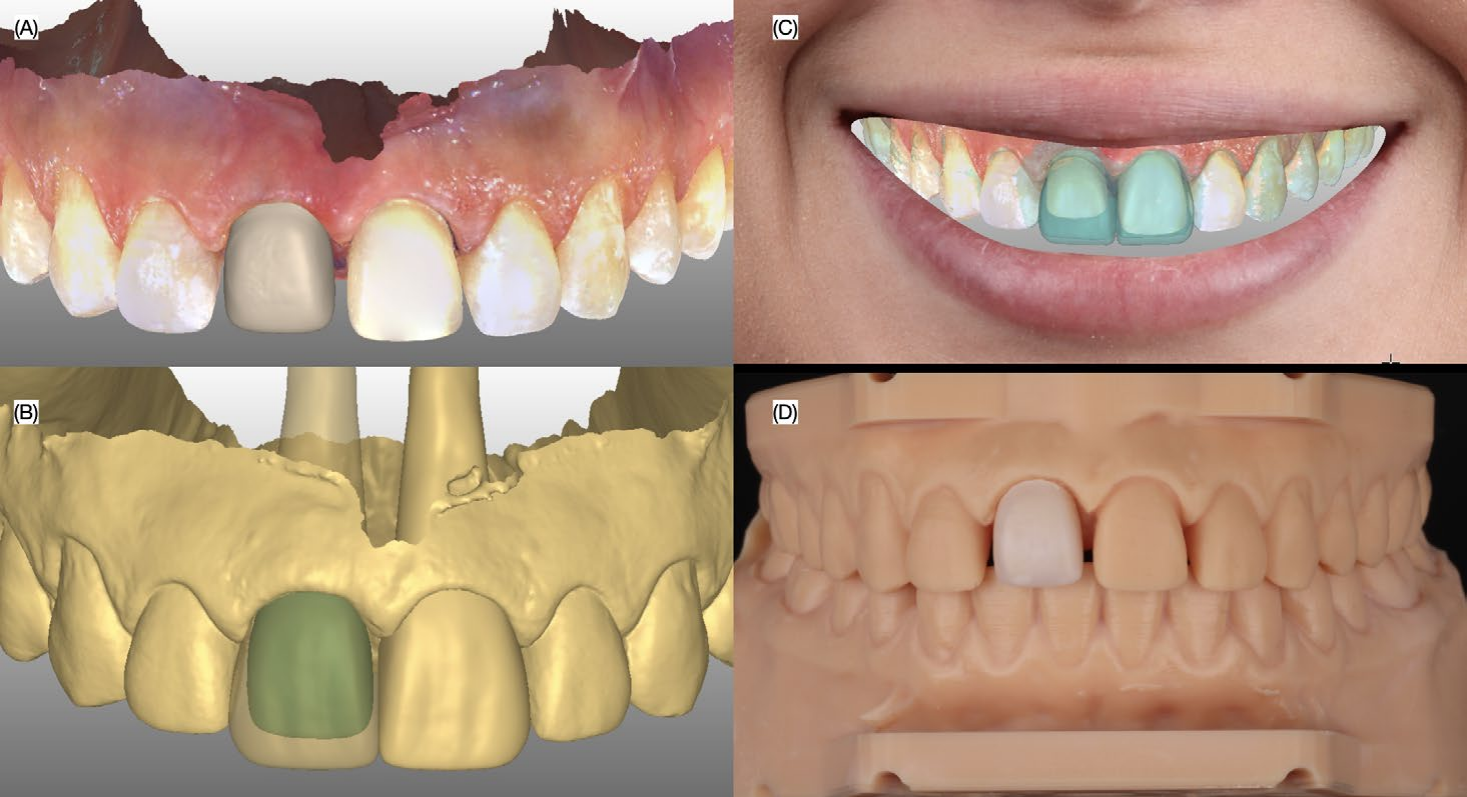
Ceramic core design and fabrication. Digital design of the ceramic core in the (A) intraoral scan, (B) digital model, and (C) smile, and (D) milled core and placed in a printed model.

Intraoral photographs were captured with a photo calibration card (Matisse Photo Calibration Card, Smile Line USA, Wheat Ridge, CO, USA) and shade tables (3 Dispersive Shade Guide, Zirkonzahn, Gais, Italy) to accurately match the shade of the final restorations (Figure 5). This data was used to digitally design a zirconia core (Zirkonzahn Prettau 3, Zirkonzahn, Gais, Italy) that would effectively mask the underlying discoloration and provide a matching substrate to the adjacent tooth. The design also incorporated the tentative contours for the future veneer restorations, which were shaped based on intraoral photos and the patient's smile. A 3D model was printed (Die and Model 2, SprintRay, Los Angeles, CA, USA) to evaluate the fit of the zirconia core (Figure 6). Shade selection was performed under standardized lighting conditions using natural daylight supplemented by color‐corrected LED illumination. Clinical photographs were obtained using a digital camera with fixed settings (ISO, aperture, and shutter speed) to ensure consistency and reproducibility. Cross‐polarization was not utilized in this case.

**FIGURE 7:**
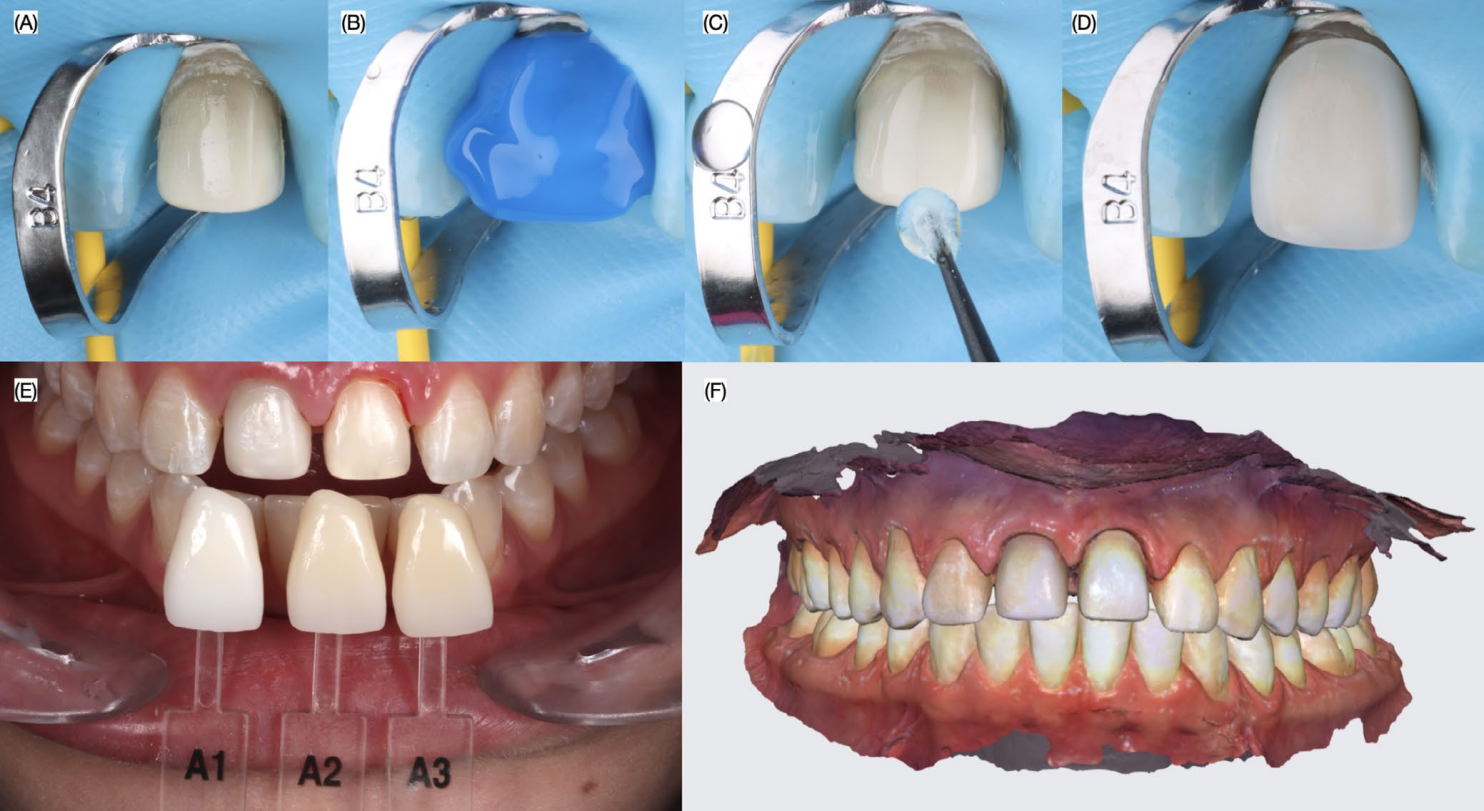
Core cementation and digital impression. (A) Isolation, (B) phosphoric acid application, (C) adhesive application, (D) core cemented, (E) photos with shade guides and (F) final digital impression for veneer restorations.

At the subsequent appointment, complete isolation was achieved using a dental dam (Nic Tone Dental Dam, MDC Dental USA, Gardena, CA, USA). A clamp (B4 Brinker, Hygenic Rubber Dam Clamps, City of Industry, CA, USA) was placed on the maxillary right central incisor to retract the soft tissue. The zirconia core was treated with 50‐μm aluminum oxide (Cobra Aluminum Oxide, Renfert GmbH, Hilzingen, Germany) at 2 bar pressure and cleaned with a cleaning paste for zirconia (Zirclean, Bisco Inc., Schaumburg, IL, USA) water in an ultrasonic bath for 5 min, aplicación de primer (Clearfil Ceramic Primer Plus, Kuraray Noritake Dental Inc., Tokyo, Japan) and then tooth surface was treated with 29‐μm aluminum oxide (AquaCare, Velopex International, London, United Kingdom) was etched with 37% phosphoric acid (Ultra‐Etch, Ultradent, South Jordan, UT, USA) for 20 s. Adhesive (All Bond Universal, Bisco Inc., Schaumburg, IL, USA) was applied and air‐thinned, and the core was cemented using resin cement (Panavia V5, Kuraray, Tokyo, Japan). Excess cement was removed, and each surface facial, mesial, distal, and incisal was light‐cured for 20 s. Additional intraoral photographs were taken with shade tabs to confirm the final shade for the veneers, and a new intraoral scan was obtained (Figure 7).

**FIGURE 8:**
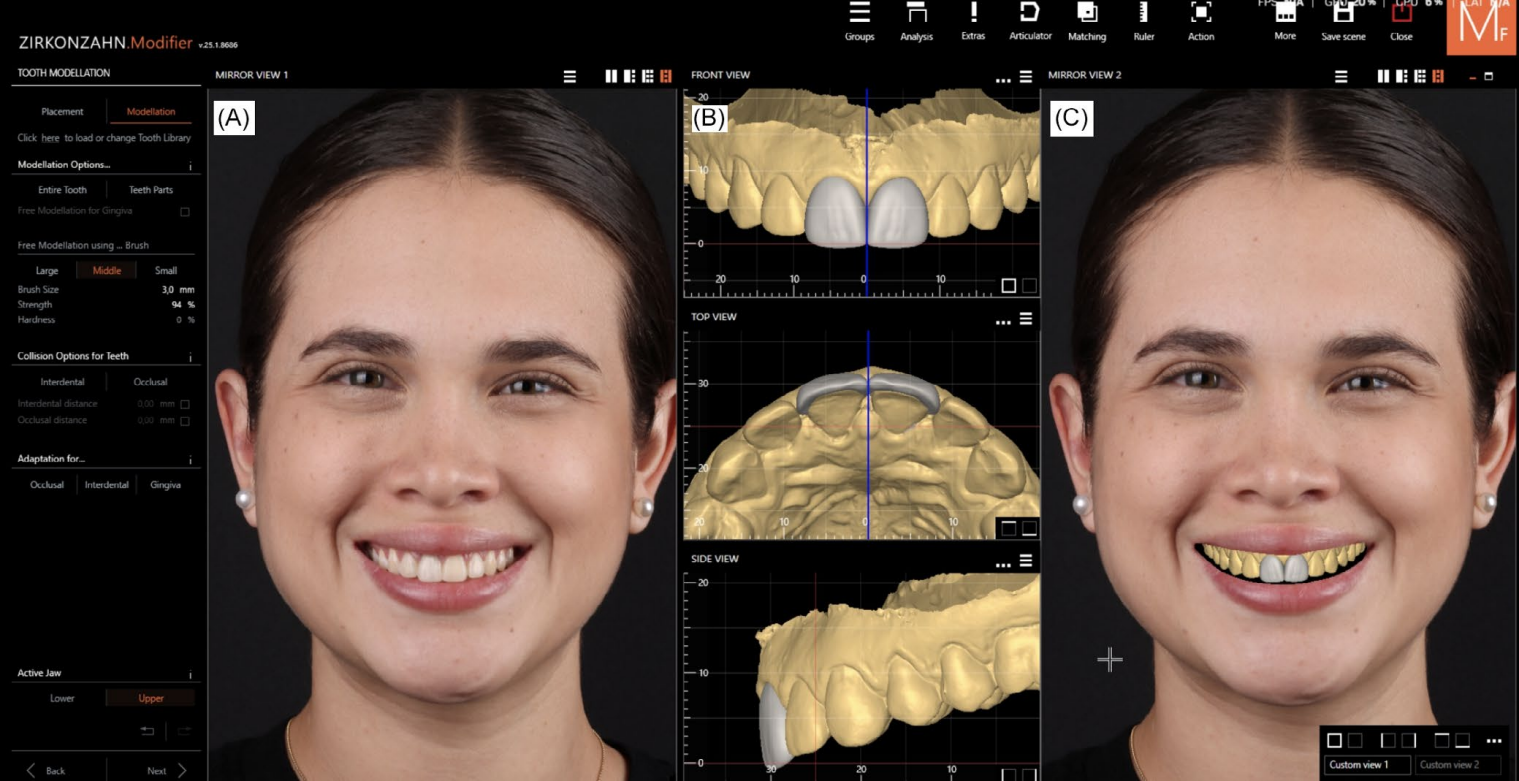
Digital design of veneer restorations. (A) Face smiling with provisional restorations, (B) digital mode with the design of veneers, and (C) face smiling with the proposed design of the veneers.

**FIGURE 9:**
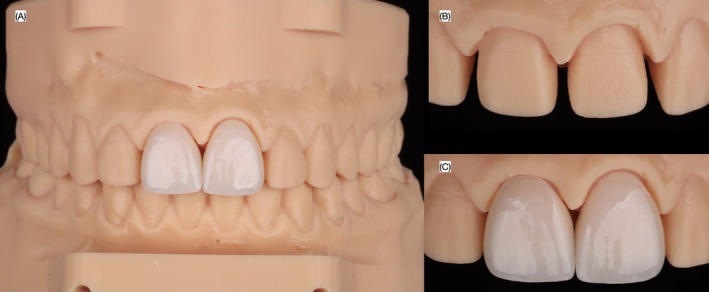
Fabrication of the ceramic veneers. (A) Printed model with both veneers in occlusion, and close up of the model (B) without and (C) with the veneers.

**FIGURE 10:**
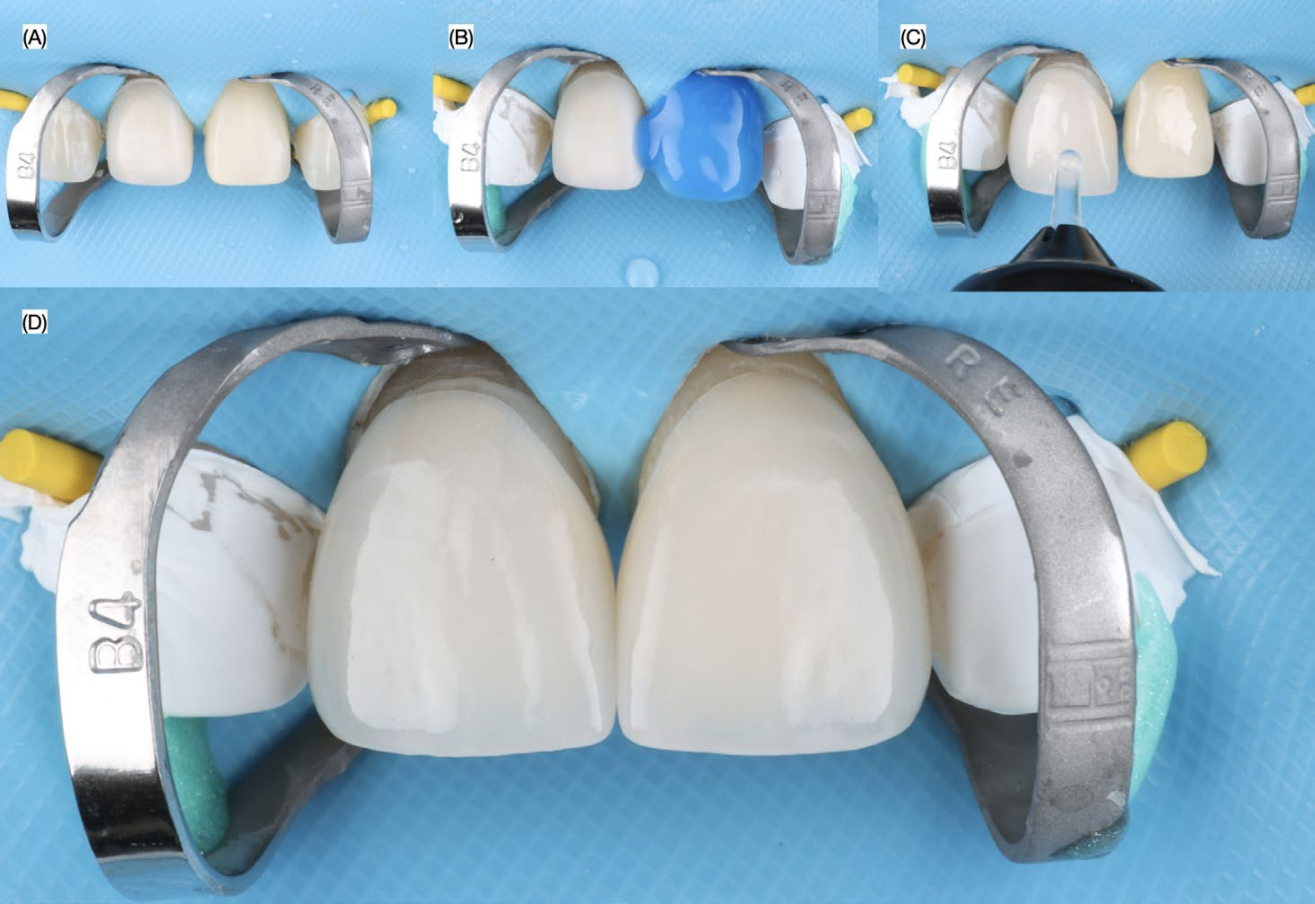
Bonding ceramic veneers. (A) Dental dam isolation and clamps placement, (B) phosphoric acid on maxillary right central incisor, (C) placement of the veneer on core and (D) placement of both veneers.

Veneer restorations were digitally designed (Zirkonzahn Modellier, Zirkonzahn Gais, Italy), with contours customized to the patient's smile. The veneers were milled from highly translucent, lithium disilicate (A2 Zirkonzahn Prettau 4 Disperse, Zirkonzahn, Gais, Italy) and a model was printed to evaluate the contours before final cementation (Figures 8 and 9). At the cementation appointment, a rubber dam (Nic Tone Dental Dam, MDC Dental USA, Gardena, CA, USA) was placed along with retracting clamps (B4 Brinker, Hygenic Rubber Dam Clamps, City of Industry, CA, USA) on both central incisors. A dry try‐in was performed to evaluate the fit and margins. The ceramic core on the maxillary right central incisor was sandblasted with 29‐μm aluminum oxide (AquaCare, Velopex International, London, United Kingdom) cleaned using a ceramic cleaning paste (Zirclean, Bisco Inc., Schaumburg, IL, USA) for 20 s, rinsed, and air‐dried. Then ceramic primmer was applied (Clearfil Ceramic Primer Plus, Kuraray Noritake Dental Inc., Tokyo, Japan) and the excess was removed with air. Then the maxillary left central incisor tooth surface was also treated with 29‐μm aluminum oxide (AquaCare, Velopex International, London, United Kingdom). Then tooth surface was etched with phosphoric acid (Ultra‐Etch, Ultradent, South Jordan, UT, USA) for 20 s, rinsed, and air‐dried, followed by the application of adhesive (All Bond Universal, Bisco Inc., Schaumburg, IL, USA). Both veneers were cemented with resin cement (Panavia Veneer LC, Kuraray, Tokyo, Japan), and excess was removed with a microbrush. Each surface of both restorations was light‐cured for 20 s (Figure 10).

**FIGURE 11:**
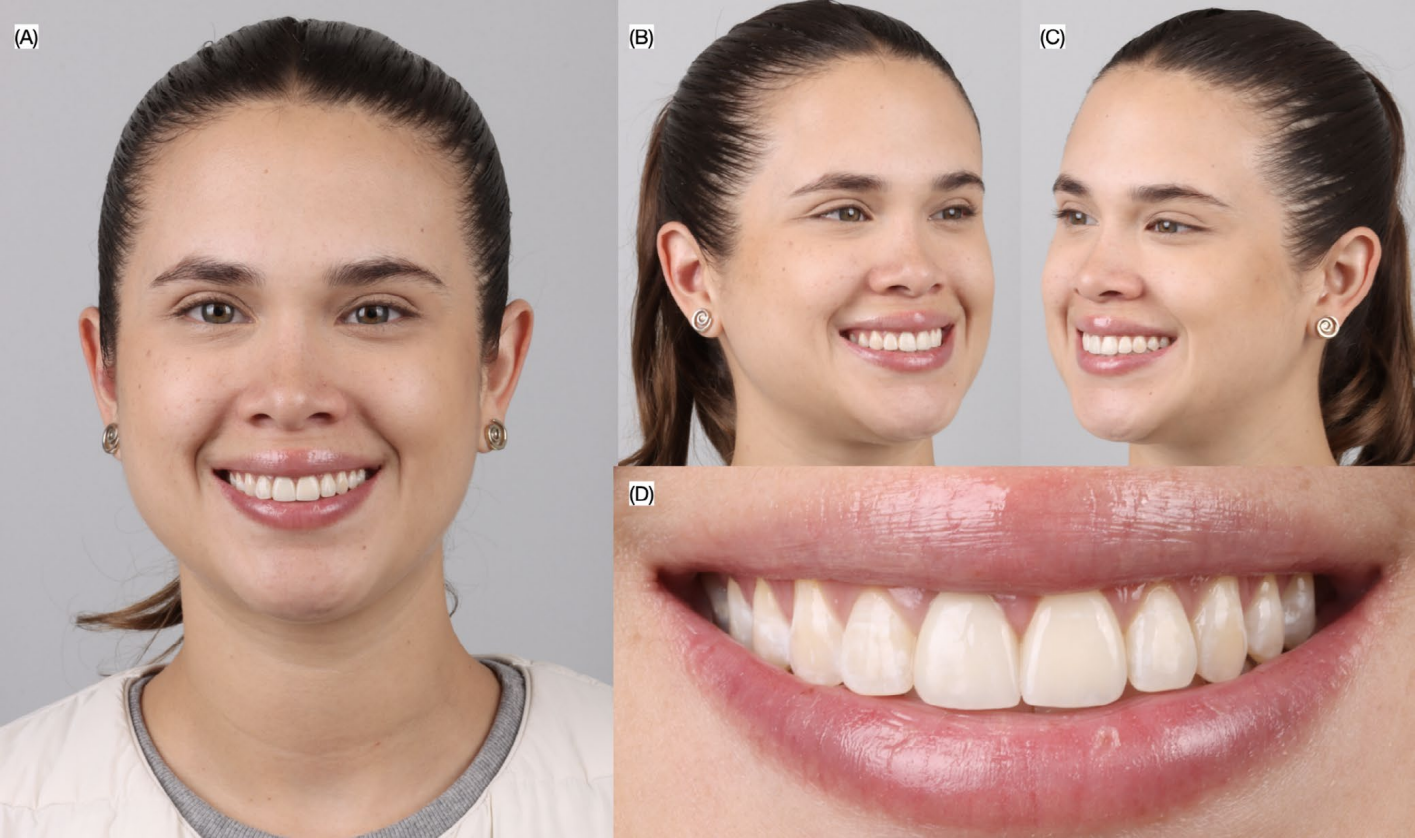
Final smile. (A) Frontal, (B) left and (C) right of face smiling and (D) close‐up of the smile.

**FIGURE 12:**
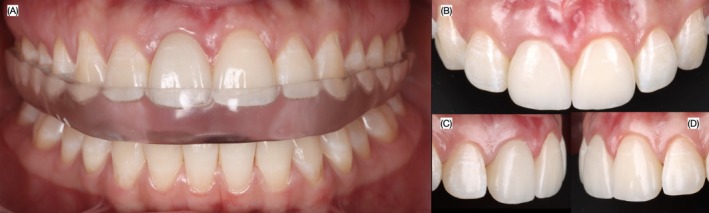
Final intra‐oral. (A) Veneers in occlusion with night guard, (B) frontal, (C) right and (D) left side view.

**FIGURE 13:**
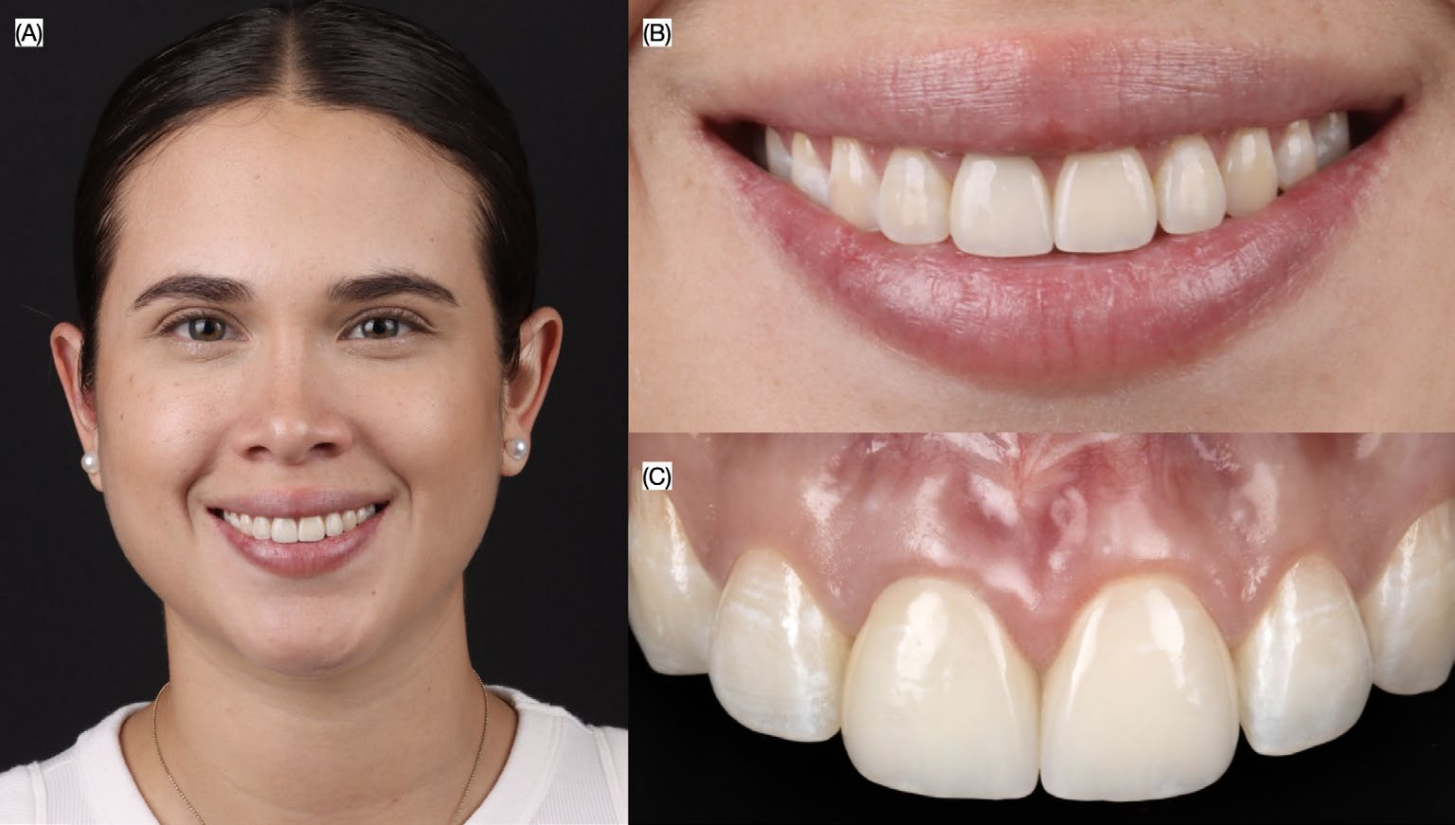
Two years follow‐up. (A) Face smiling, (B) close up of the smile, and (C) intraoral.

**FIGURE 14:**
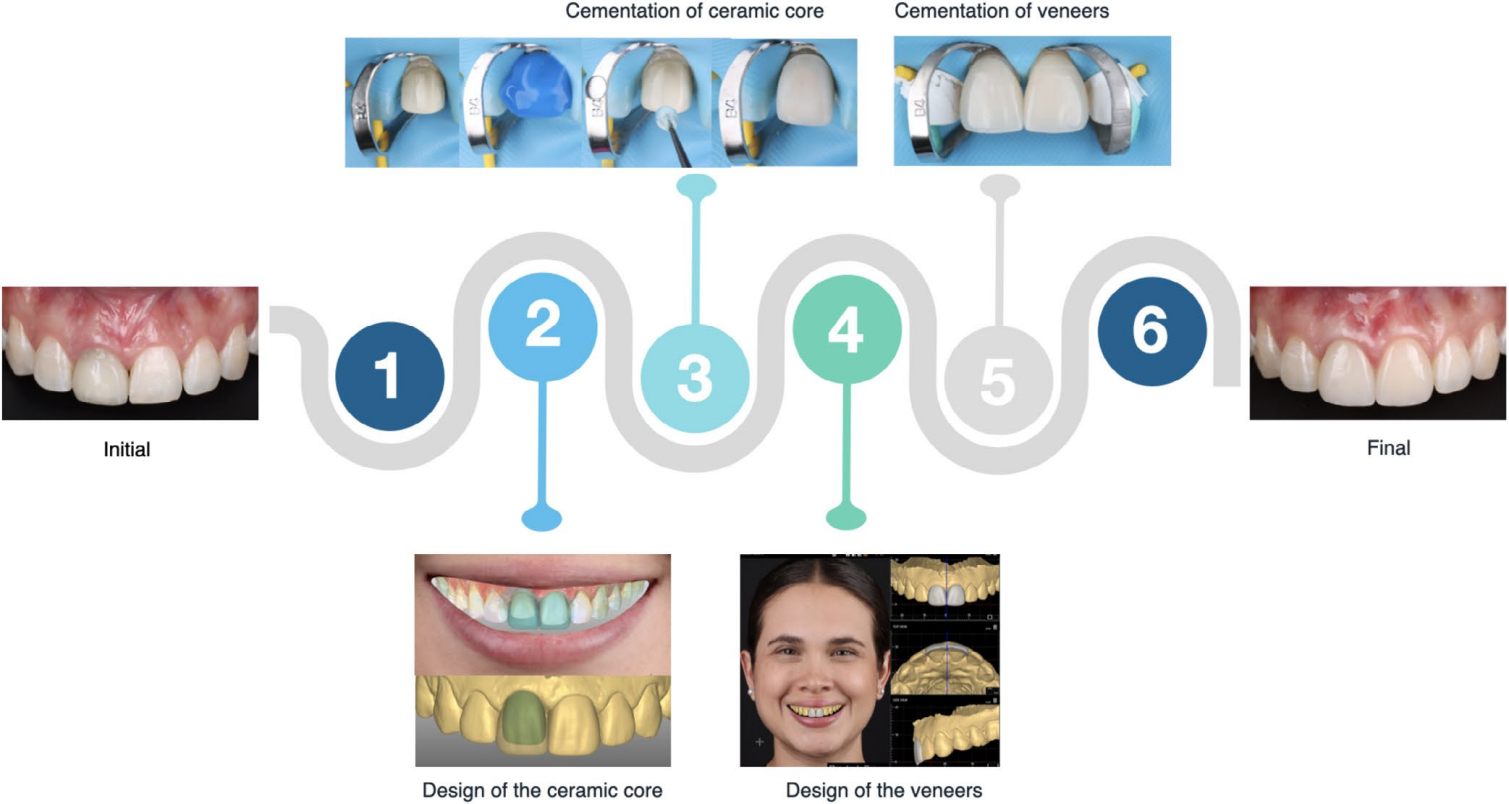
Digital workflow performed in this case study. (1) Initial situation, (2) digital design of the ceramic core, (3) bonding of the ceramic core, (4) digital design of the veneers, (5) cementation of the veneers under total isolation with dental dam and (6) final result.

Occlusion was verified in maximum intercuspation, excursive movements, and protrusive contacts. The patient expressed satisfaction with the contours, shade, and shape of the final restorations, as well as with the overall improvement of her smile. An occlusal guard was provided to protect the restorations during nighttime use (Figures 11 and 12). At the 2‐year follow‐up, the ceramic restorations and surrounding gingival tissues remained in excellent condition (Figure 13). The clinical workflow detailing each step of the treatment process is summarized in Figure 14.

## 4 Discussion

The treatment of stained teeth in the esthetic zone can present a challenging clinical scenario. Accurate diagnosis is essential, as stains are typically classified as either extrinsic or intrinsic. Extrinsic stains appear on the external surface of the tooth and are often caused by poor oral hygiene, consumption of pigmented foods and beverages, or tobacco use. These stains can usually be removed through routine prophylactic procedures, although darker stains may require whitening treatments [14]. In contrast, intrinsic stains are more difficult to treat, as they originate from within the tooth structure. Common causes include aging, tetracycline use, dental caries, or prior restorations. Discoloration from medications may occur either before or after tooth formation. Intrinsic stains generally cannot be removed through standard cleaning but may be reduced with whitening treatments. Depending on the severity of the discoloration, treatment options may range from tooth whitening to restorative procedures designed to fully mask the stain [15]. The present case involved intrinsic staining from previous endodontic treatment, which posed a significant challenge and required a more complex restorative approach.

Ceramic restorations such as veneers may be used to mask stained teeth in the esthetic zone [16]. However, clinicians should be aware that dark discoloration may not always be completely masked by veneers. One recent in vitro study evaluated the shade‐masking ability of CAD/CAM lithium disilicate and leucite ceramics at thicknesses of 0.5 mm, 1 mm, and 1.5 mm, applied over nine shades of dentin. The study used a digital spectrophotometer to measure color and translucency differences. The results showed that the 0.5 mm specimens produced the greatest color and translucency differences, while the 1.5 mm specimens produced the least, demonstrating that increasing ceramic thickness improves masking ability and reduces translucency [17]. Moreover, a more recent systematic review of 13 in vitro studies on masking ability found that 1.5 mm lithium disilicate offered better masking performance than 1.0 mm in difficult‐to‐mask situations. The authors concluded that although darker substrates are inherently harder to mask, a thickness of 1.5 mm provides the best compromise between coverage and esthetics [18]. In the present clinical scenario, the tooth exhibited intense discoloration. Since fabricating a labial veneer with 1.5 mm thickness is uncommon and may be less practical, a ceramic core was fabricated before the veneer restoration to improve masking effectiveness.

The combination of a ceramic core and veneer restoration can help mask dark substrates and facilitate the technician's work in fabricating veneers with ideal proportions and a more predictable shade outcome. A recent in vitro study evaluated the masking properties of combined lithium disilicate core and veneer restorations at different thicknesses: 0.4/0.4 mm, 0.5/0.5 mm, 0.6/0.6 mm, 0.8/0.7 mm, 1.0/0.8 mm, and 1.1/0.9 mm. Using a spectrophotometer to measure color, the results showed that 0.8/0.7 mm and 1.0/0.8 mm combinations achieved clinically acceptable color differences in masking a black background. The authors concluded that a total thickness of 1.5 mm (core + veneer) is required to mask a dark discoloration [19]. Another in vitro study assessed different core and veneer thicknesses from lithium disilicate and leucite ceramics: cores of 0.8 mm, 1.0 mm, and 1.2 mm paired with veneers of 0.7 mm, 0.5 mm, and 0.3 mm. They evaluated translucency, light transmittance, contrast ratio, and spectral reflectance via spectrophotometry, finding that the combination of a 1.2 mm core with a 0.3 mm veneer produced the most stable and least variable color values. The authors concluded that final color outcomes in ceramic core–veneer combinations are significantly influenced by thickness [20]. In this clinical report, a 1.0 mm core and 0.5 mm veneer were chosen, in line with prior studies recommending a total thickness of around 1.5 mm for effective masking.

Gingivectomy is a surgical procedure involving the removal of unsupported gingival tissue to create a new gingival margin in a more apical position [21, 22]. This procedure is typically performed to enhance the esthetic architecture of the gingiva and can be carried out using a scalpel, electrosurgery, laser, or chemosurgery [23]. For teeth with probing depths of ≥ 4 mm, gingivectomy is recommended. However, if there is insufficient keratinized gingiva, crown lengthening is preferred, as osseous recontouring may be necessary to reestablish the biologic width at a more apical level [24]. The literature suggests a healing period of approximately 4–6 weeks [25, 26]. In the present case, the patient exhibited a probing depth of 4 mm. As a result, a gingivectomy was performed with the removal of 2 mm of soft tissue, followed by a healing period of 6 weeks prior to proceeding with further restorative treatment.

Bonding ceramic restorations under full isolation with a dental dam is strongly recommended in the literature [27, 28]. Using a dental dam provides multiple benefits: it improves visibility by retracting surrounding tissues, ensures aseptic isolation to prevent contamination from saliva and blood, and protects the patient's airway from accidental ingestion or aspiration of instruments, irritants, and debris, while also reducing aerosol contamination especially relevant in the post‐COVID era [29]. However, clinicians must confirm that the patient is not allergic to latex; in such cases, non‐latex alternatives should be used [30]. In this clinical scenario, a dental dam was applied during bonding of both the ceramic core and veneer restorations. Its use also supports more predictable adhesive performance by maintaining a completely dry field throughout the procedure. At the 2‐year follow‐up, the restorations demonstrated stable clinical performance. No evidence of debonding, marginal discoloration, chipping, or fracture was observed. The patient reported no postoperative sensitivity or discomfort.

## 5 Conclusion

The clinical findings align with previous studies showing that combining a ceramic core and veneer restoration can successfully mask dark stained teeth. Compared to fabricating a single thick veneer, using a core and regular‐dimensioned veneers facilitates the creation of highly esthetic restorations. A multidisciplinary approach including gingivectomy and ceramic veneers helped fulfill the patient's esthetic demands. The digital workflow, especially the digital design of the final restorations, allowed evaluation of outcomes before actual fabrication, improving predictability.

## Author Contributions

**Francisco Garcia‐Torres:** conceptualization, methodology, writing – original draft. **Enrico Steger:** conceptualization, methodology. **Alexander Lichtmannegger:** conceptualization, methodology. **Carlos A. Jurado:** supervision, writing – original draft, writing – review and editing. **Silvia Rojas‐Rueda:** supervision, writing – original draft, writing – review and editing. **Franciele Floriani:** writing – review and editing.

## Funding

The authors have nothing to report.

## Consent

Written informed consent was obtained from the patient for the use of clinical photographs and publication of this case.

## Conflicts of Interest

The authors declare no conflicts of interest.

## Data Availability

All data supporting the findings of this clinical report are available from the corresponding author upon reasonable request.
